# Park proximity and all-day and time-specific physical activity and sedentary behaviour in older adults

**DOI:** 10.1186/s12877-024-05527-8

**Published:** 2024-11-13

**Authors:** Chien-Yu Lin, Ting-Fu Lai, Chin-Yi (Fred) Fang, Ming-Chun Hsueh, Yung Liao

**Affiliations:** 1 Department of Public Health, College of Public Health, China Medical University, Taichung, Taiwan; 2 Centre for Urban Transitions, Swinburne University of Technology, Melbourne, Australia; 3 Faculty of Sport Sciences, Waseda University, Tokorozawa, Japan; 4 Department of Health Promotion and Health Education, National Taiwan Normal University, Taipei, Taiwan; 5 Health Convergence Medicine Laboratory, Biomedical Research Institute, Pusan National University Hospital, Busan, South Korea; 6 Graduate Institute of Sport, Leisure and Hospitality Management, College of Sports and Recreation, National Taiwan Normal University, 6F, No 129-1, Section 1, Heping East Road, Da’an District, Taipei City, 106 Taiwan; 7 Graduate Institute of Sport Pedagogy, University of Taipei, Taipei, Taiwan; 8 Master’s Program of Transition and Leisure Education for Individuals with Disabilities, University of Taipei, Taipei, Taiwan

**Keywords:** Built environment, Open space, Circadian rhythm, Step, Sitting, Elderly

## Abstract

**Background:**

Proximity to parks has been suggested as a factor influencing physical activity in older adults. However, it remains unclear the optimal distance between residences and parks for promoting physical activity and reducing sedentary time and whether these associations vary by the time of day. We examined whether the proximity to neighbourhood parks at varying distances is associated with all-day and time-specific physical activity and sedentary behaviour in older adults.

**Methods:**

Data were collected from 214 older adults receiving hospital services in Taipei, Taiwan. The number of parks within 400m, 800m, and 1,600m of participants’ residences. Physical activity and sedentary behaviour, stratified by time of day (morning, afternoon, and evening), were measured using accelerometers. Adjusted linear regression models were used to estimate associations of park proximity with activity and sedentary outcomes.

**Results:**

Parks located within 400m and 800m of participants’ residences were more markedly associated with longer time in physical activity and less sedentary time compared to parks located 1,600m away. A greater number of parks within 400m and 800m was positively associated with walking steps and light-intensity physical activity while both distances were negatively associated with sedentary time. The associations between park proximity and behavioural outcomes were mainly attributable to that during the afternoon and evening.

**Conclusions:**

Our findings suggest that favourable access to parks within 800m of older adults’ residences is associated with more physical activity and less sedentary time, particularly during the afternoon and evening. Future longitudinal studies are required to corroborate these associations.

## Introduction

Physical inactivity and a high amount of sedentary time are global public health priorities. In 2008, a lack of physical activity accounted for nearly 10% of premature mortality globally [15] and was estimated to cost healthcare systems INT$ 53.8 billion in 2013 [9]. A meta-analysis suggested that at least 8% of all deaths can be prevented by eliminating prolonged sedentary behaviours [20], potentially saving approximately 70,000 lives in England alone [11]. Therefore, initiatives aimed at increasing physical activity and reducing sedentary time are crucial.

Among the potentially influential factors of physical inactivity and prolonged sedentary time, there is growing interest in the availability of public open spaces near residential areas [7, 12, 14, 34]. Systematic reviews have found that closer proximity to and a greater number of public open spaces are associated with higher levels of recreational physical activity across all age groups [2, 17]. Another review focused on adults aged 18–64 years also found that greater proximity to and higher density of green spaces was linked to reduced sedentary behaviour [19]. These relationships may be more pronounced in older adults, who generally spend more time at home and experience higher exposure to their residential neighbourhoods. However, studies targeting older populations have yielded mixed and inconclusive findings regarding the associations between park proximity and physical activity and sedentary behaviour. For instance, a Hong Kong study showed that park availability was associated with increased recreational walking [5], while a Dutch study found positive associations between the number of parks and moderate-to-vigorous-intensity physical activity (MVPA) but not light-intensity physical activity (LPA) [22]. By contrast, a previous study from Hong Kong indicated no association between the presence of parks and non-transport sitting or motorised transport [3], whereas a study from the US showed that frequent visits to local park were associated with higher levels of physical activity [18]. Furthermore, the optimal distance between residences and parks for influencing physical activity in older adults remains unclear.

Evidence suggests that patterns of park visitation vary by time of day [6, 24]. Temporal differences in park usage may cause differences in the associations between park proximity and behaviours. Proximity to parks may be relatively important when people access parks and engage in physical activities or sedentary behaviours within parks, or when neighbourhood parks serve as a primary places for travel. Several observational studies have identified important features within neighbourhood parks that contribute to users’ physical activity and sedentary behaviour throughout the day [25, 36]. A Chinese study found that more people visited neighbourhood parks in the morning than in the afternoon or noon [36]. Another US study also showed similar patterns in urban areas, while rural areas showed the contrary pattern, with the lowest number of park visits in the morning [25]. Notably, residents could also benefit from neighbourhood parks [2, 17]; travelling to or from neighbourhood parks or using neighbourhood parks as a shortcut for commuting may contribute to framing overall activities for residents who did not use features within parks.

We aimed to investigate the associations of the number of parks in participants’ residential neighbourhoods (i.e., an indicator for park proximity) with all-day and time-specific physical activities and sedentary time in a sample of city-based older residents aged 65 years or above.

## Methods

### Participants

Older adults aged 65 years or above were recruited from geriatrics and gerontology outpatient clinics and community check-up sites in Taipei City, the capital of Taiwan, from September 2020 to March 2021. Potential participants, who had basic conversational abilities, were primarily screened by physicians or research assistants to ensure they could understand the study procedures and instructions. Participants were requested to complete a questionnaire with regard to individuals’ sociodemographic characteristics (e.g., age, gender, and living status), self-reports of health conditions (e.g., hypertension, hyperlipidaemia, and diabetes), and residential addresses (*n* = 277). They were also instructed to wear an accelerometer on their hip for seven consecutive days. Each participant signed an informed consent prior to each start of the survey. A convenience store voucher valued at USD 7 was provided to participants who completed all study requirements. This study was approved by the Research Ethics Committee of the National Taiwan University Hospital (202008046RINC).

### Exposure: proximity to neighbourhood parks

Park proximity was assessed by the number of parks within 400m, 800m, and 1,600m of participants’ residences [37], using geographic information systems (GIS). Parks were defined as public open spaces designated for activities and recreation and managed by local authorities. Park locations were obtained from the National Land Surveying and Mapping Center, Ministry of the Interior. These park locations were visualised using GIS based on the coordinates and calculated the number of parks within specific residential buffers of each individual. A greater number of parks within a neighbourhood indicates greater proximity to parks. The examination of different residential buffers can help identify the influential residential neighbourhoods for older adults in accessing parks. All these GIS-based measures were analysed using the ESRI ArcGIS Desktop10 software package.

### Outcomes: the time of physical activities and sedentary behaviour

Physical activity and sedentary behaviour were measured using an accelerometer (model wGT3X-BT; ActiGraph, Pensacola, FL, USA), which has demonstrated validity and reliability [29]. Participants wore the accelerometer on the right side of their waist for seven consecutive days. They were instructed to remove the accelerometer only for water-based activities (e.g., showering and swimming). Participants who provided accelerometer data ≥ 10 h/day and ≥ four days (including at least one weekend day) were considered to have provided eligible data.

Accelerometers measure vibration by monitoring acceleration and converting it into voltage. Data from the accelerometer were categorised into sedentary (≤ 99 counts/minute), LPA (100-2,019 counts/minute), and MVPA (≥ 2,020 counts/minute) [29]. For physical activities, we assessed total step counts, LPA, and MVPA. In terms of sedentary behaviour, we assessed the total time in all kinds of sedentary behaviours. The all-day and time-specific amounts of physical activities and sedentary behaviour were calculated. Three time segments were identified, including morning (06:00 am-12:00 pm), afternoon (12:01 pm-06:00 pm), and evening (06:01 pm-12:00 am), aligning with previous studies [13, 23]. Participants’ bedtimes and wake times were recorded in a sleep log and used to identify non-wear time [16, 30]. All accelerometer data with 60-second epochs were processed using the ActiLife software 6.0 (Pensacola, FL, USA) [1].

### Covariates

We included sociodemographic characteristics i.e., age (in years), gender (men or women), living status (alone or with others), and educational level (university and above or not), health status (i.e., body mass index [BMI], hypertension, hyperlipidaemia, diabetes, cognitive function, and nutritional status), and health behaviours (i.e., drinking and smoking) as covariates. All these variables were collected using a questionnaire. BMI was calculated as self-reported weight (in kilograms) divided by the square of height (in metres). Participants were requested to report if they had been diagnosed or taken medicine for hypertension, hyperlipidaemia, and diabetes (yes/no) [31]. The Mini-Mental State Examination [28] and Mini Nutritional Assessment [10], which were validated elsewhere, were used to assess participants’ cognitive function and nutritional status. The cut-off points for cognitive function were differently set based on the education level (≤ 16 for those non-educated, ≤ 20 for those educated elementary, and ≤ 23 for those educated junior school and above as high-risk) [28]; and that for nutritional status was consistent for all population (≤ 11 as high-risk). Drinking and smoking behaviours were identified by whether participants were current smokers and current drinkers, respectively. We also adjusted for the total accelerometer wear time in the regression models.

### Statistical analyses

The mean and standard deviation (SD) or number and percentage were presented for descriptive analyses of participants’ characteristics, exposure, and outcomes. Given the skewed distribution of outcome variables, we additionally reported the median and interquartile range (IQR), showing the values of quartiles 1 and 3. Linear regression models were used to estimate the associations between the number of parks and all-day and time-specific physical activities and sedentary behaviour across three different buffers, after adjusting for all covariates. Logistic regression models were not used in this study as we examined the patterns throughout the day, and ‘sufficient’ physical activity levels were difficult to define by time segment. Considering the skewed distribution of physical activity measures, these variables were log-transformed prior to being included in the models. Unstandardised regression coefficients (B) and their 95% confidence intervals (CIs) were estimated. We calculated the R^2^ for each model, including models for different residential buffers and behaviours during different time segments, to check the model fit. All analyses were conducted using IBM SPSS 23.0 software (SPSS Inc., IBM, Chicago, IL, USA), with the significance level set at *p* < 0.05.

## Results

### Characteristics of participants

After excluding participants who provided incomplete residential addresses that cannot be geocoded (*n* = 13) and those with invalid data on accelerometer-based behaviours (*n* = 50), 214 eligible older adults were included in the analyses. The majority of participants resided in Taipei City (69.2%), with others living in neighbouring cities, including New Taipei City (23.8%) and Taoyuan City (4.2%), which share a common living space.

Table 1 shows the characteristics of the participants. Participants were 45.8% men, with a mean age of 76.5 years and an average BMI of 24.2 kg/m^2^. Over 90% of the participants did not engage in unhealthy drinking or smoking habits and were in relatively good health. For instance, 86.9% were classified as low-risk for cognitive impairment and 79.0% were considered low-risk for nutritional deficiencies.

**Table 1:** Participants’ characteristics (*n* = 214)

Characteristics	Mean (SD) or *n* (%)
Age (years)	76.5 (6.7)
Gender	
Men	98 (45.8%)
Women	116 (54.2%)
Living status	
Living with others	194 (90.7%)
Living alone	20 (9.3%)
Educational level	
Lower than university	112 (52.3%)
University and above	102 (47.7%)
BMI (kg/m^2^)	24.2 (3.6)
Drinking	
No	193 (90.2%)
Yes	21 (9.8%)
Smoking	
No	198 (92.5%)
Yes	16 (7.5%)
Hypertension	
No	85 (39.7%)
Yes	129 (60.3%)
Hyperlipidaemia	
No	117 (54.7%)
Yes	97 (45.3%)
Diabetes	
No	139 (65.0%)
Yes	75 (35.0%)
Cognitive function	
Low-risk	186 (86.9%)
High-risk	28 (13.1%)
Nutritional status	
Low-risk	169 (79.0%)
High-risk	45 (21.0%)

SD: standard deviation

Table 2 summarises the descriptive analyses of the park proximity and accelerometer-based physical activity and sedentary time across all-day and time-specific periods. On average, participants accumulated approximately 5,300 steps per day, of which mainly occurred in the morning and afternoon, more than twice as many counts as in the evening. Similar patterns were observed for MVPA. By contrast, participants spent most of their time engaging in LPA and being sedentary in the afternoon, accounting for 41.0% and 38% of all-day LPA and sedentary behaviour, respectively. Of note, half of the participants engaged in a low amount of MVPA (< 10 min per day).

**Table 2:** Summary of GIS-based number of parks, by the buffer, and physical activities and sedentary time, all day and time of the day

	Mean	SD	Median (IQR)	% of overall behaviours
Number of parks				
400-m buffer	3.4	2.4	3.0 (1.0–5.0)	-
800-m buffer	12.4	5.9	13.0 (8.0–17.0)	-
1,600-m buffer	44.5	16.2	44.0 (38.0-54.8)	-
Step counts (counts/day)				
All-day	5347.0	3693.8	4908.0 (2674.3-7123.1)	100.0
Morning	2182.0	1886.0	1513.0 (801.6-3337.9)	40.6
Afternoon	2145.7	1648.8	1719.8 (877.4-3026.3)	39.9
Evening	1046.0	1239.3	660.6 (321.2-1327.8)	19.5
LPA (minutes/day)				
All-day	244.3	86.2	238.0 (184.8-286.5)	100.0
Morning	70.3	50.4	71.4 (35.7-103.9)	31.4
Afternoon	91.9	34.9	88.7 (67.9-110.2)	41.0
Evening	62.0	32.0	55.4 (39.4–77.3)	27.7
MVPA (minutes/day)				
All-day	15.5	24.0	7.2 (1.3–21.4)	100.0
Morning	6.3	10.9	1.7 (0.3–8.2)	41.4
Afternoon	6.1	10.0	2.2 (0.3–7.2)	40.2
Evening	2.8	8.2	0.7 (0.2–2.3)	18.4
Sedentary time (minutes/day)				
All-day	608.1	79.2	615.1 (561.4-657.5)	100.0
Morning	181.5	51.3	183.3 (149.6-219.2)	30.3
Afternoon	227.7	33.0	230.6 (206.4-250.2)	38.0
Evening	190.5	46.7	193.6 (160.8–220.0)	31.8

SD, standard deviation; IQR, interquartile range; LPA, light-intensity physical activity; MVPA, moderate-to-vigorous-intensity physical activity; PA, physical activity

### Associations of the number of parks with time in physical activities and sedentary behaviour

The associations of the number of parks with time of physical activities and sedentary behaviour were more pronounced when the residential buffer was set as 400m and 800m compared to 1,600m (Table 3). The model fit, indicated by R^2^, was higher for all-day step counts (R^2^ = 0.46–0.48) and LPA (R^2^ = 0.47–0.48) than for MVPA (R^2^ = 0.23–0.24) and sedentary time (R^2^ = 0.20–0.22); the value of R^2^ decreased as the buffer extended.

**Table 3:** Associations of the number of parks with all-day and time-specific step counts, physical activities, and sedentary time in older adults

	400-m buffer							800-m buffer							1,600-m buffer					
	B	(95% CI)			p	R^2^		B	(95% CI)			p	R^2^		B	(95% CI)			p	R^2^
^a^ Step counts																				
All-day	**0.06**	**(0.02**	,	**0.09)**	**0.002**	**0.48**		**0.02**	**(0.01**	,	**0.04)**	**0.003**	**0.48**		0.00	(0.00	,	0.01)	0.14	0.46
Morning	0.05	(-0.01	,	0.10)	0.075	0.37		0.02	(0.00	,	0.04)	0.11	0.37		0.00	(-0.01	,	0.01)	0.46	0.36
Afternoon	**0.06**	**(0.02**	,	**0.10)**	**0.008**	**0.40**		**0.03**	**(0.01**	,	**0.04)**	**0.005**	**0.40**		**0.01**	**(0.00**	,	**0.01)**	**0.046**	**0.39**
Evening	**0.09**	**(0.04**	,	**0.14)**	**< 0.001**	**0.30**		**0.03**	**(0.01**	,	**0.05)**	**0.005**	**0.28**		**0.01**	**(0.01**	,	**0.02)**	**< 0.001**	**0.30**
^a^ LPA																				
All-day	**0.02**	**(0.01**	,	**0.04)**	**0.007**	**0.48**		**0.01**	**(0.00**	,	**0.01)**	**0.043**	**0.47**		0.00	(0.00	,	0.00)	0.27	0.47
Morning	0.16	(-0.45	,	0.77)	0.60	0.06		0.09	(-0.17	,	0.35)	0.50	0.08		0.10	(-0.01	,	0.20)	0.061	0.06
Afternoon	**0.02**	**(0.00**	,	**0.04)**	**0.045**	**0.38**		0.01	(0.00	,	0.02)	0.055	0.38		0.00	(0.00	,	0.01)	0.076	0.38
Evening	**0.04**	**(0.01**	,	**0.07)**	**0.004**	**0.23**		**0.01**	**(0.00**	,	**0.02)**	**0.047**	**0.21**		**0.01**	**(0.00**	,	**0.01)**	**0.001**	**0.24**
^a^ MVPA																				
All-day	0.15	(-0.08	,	0.38)	0.20	0.24		0.07	(-0.03	,	0.17)	0.18	0.24		0.00	(-0.035	,	0.041)	0.86	0.23
Morning	0.12	(-0.33	,	0.57)	0.59	0.27		0.12	(-0.08	,	0.31)	0.24	0.27		0.04	(-0.036	,	0.111)	0.32	0.27
Afternoon	0.39	(-0.05	,	0.82)	0.90	0.23		**0.22**	**(0.04**	,	**0.40)**	**0.019**	**0.24**		0.04	(-0.035	,	0.108)	0.31	0.23
Evening	0.42	(-0.12	,	0.97)	0.12	0.10		0.23	(0.00	,	0.46)	0.054	0.11		0.04	(-0.049	,	0.131)	0.37	0.09
Sedentary time																				
All-day	**-5.00**	**(-9.16**	,	**-0.84)**	**0.019**	**0.22**		**-2.00**	**(-3.77**	,	**-0.24)**	**0.026**	**0.22**		-0.31	(-0.99	,	0.37)	0.37	0.20
Morning	-0.45	(-2.37	,	1.47)	0.64	0.14		-0.44	(-1.29	,	0.40)	0.30	0.14		0.01	(-0.34	,	0.36)	0.94	0.15
Afternoon	**-2.74**	**(-4.41**	,	**-1.06)**	**0.002**	**0.14**		**-1.03**	**(-1.74**	,	**-0.31)**	**0.005**	**0.12**		-0.27	(-0.55	,	0.00)	0.05	0.10
Evening	**-2.35**	**(-3.87**	,	**-0.82)**	**0.003**	**0.10**		**-0.79**	**(-1.44**	,	**-0.13)**	**0.019**	0.09		-0.16	(-0.43	,	0.10)	0.22	0.11

B: Unstandardised regression coefficient. Models were adjusted for age, gender, educational level, living status, BMI, hyperlipidaemia, hypertension, diabetes, cognitive function, nutritional status, alcohol, smoking, and accelerometer wear time. 95% CIs that did not include zero were highlighted in bold

^a^ Variables were log-transformed prior to the analyses

Results for park proximity within the 400m and 800m buffers were very similar. Within the 400m buffer, greater park proximity was associated with more step counts (B = 0.06; 95% CI 0.02, 0.09), longer minutes in LPA (B = 0.02, 95% CI 0.01, 0.04), and fewer minutes in sedentary behaviour (B=-5.00, 95% CI -9.16, -0.84) in a day. These associations were mainly attributable to the changes in the afternoon and evening. Within the 800m buffer, greater park proximity was also found to be associated with more step counts (B = 0.02; 95% CI 0.01, 0.04), higher minutes in LPA (B = 0.01; 95% CI 0.00, 0.01), and less sedentary time (B=-2.00; 95% CI -3.77, -0.24) in a day, contributed by changes in the afternoon and evening, although the increase in LPA in the afternoon was marginally significant (*p* = 0.055). Of note, park proximity within the 800m buffer was specifically associated with more MVPA time in the afternoon (B = 0.22; 95% CI 0.04, 0.40).

In terms of the 1600m buffer, most associations observed in the shorter buffer were largely attenuated. No associations were observed for any behaviours across the whole day; however, an increase in park proximity was still found to be associated with higher step counts during the afternoon (B = 0.01; 95% CI 0.00, 0.01) and evening (B = 0.01; 95% CI 0.01, 0.02) and more time in LPA in the evening (B = 0.01; 95% CI 0.00, 0.01).

## Discussion

### Main findings

The study results showed that the associations of physical activities and sedentary behaviour with the number of parks were more marked within 400m and 800m than within 1,600m, suggesting that the influential residential neighbourhood for older adults does not exceed 800m. A greater park proximity in the neighbourhoods was associated with more walking steps, higher levels of LPA, and less sedentary time, particularly in the afternoon and evening.

### Comparisons with previous research findings

Our results suggested the potentially influential neighbourhood for older adults’ park-related physical activity, including physical activity occurring in parks and travelling to parks, does not exceed 800m, which is smaller than that suggested for adults’ walking behaviours. An Australian study indicated that environmental correlates within buffer sizes from 600 to over 2,000 m could support adults’ walking to and from destinations such as services and natural features [27]. The differences in these findings may be attributable to the target population and study settings. A systematic review points out that residential neighbourhoods may be smaller for the older population due to the increase in functional limitations and the fear of moving outdoors [32]. The setting of this study included mostly cities (i.e., Taipei City and neighbouring cities), where population density is high and services are more accessible, while the previous Australian study collected data from urban to regional areas.

It seemed that the potentially influential residential buffers for physical activity differed by the intensity of physical activity. We found that time spent in LPA was associated with the number of parks at a shorter distance to the residence, while walking steps were associated with that at an extended distance. It may be implied that the parks well equipped with attractive walking paths and spaces for walking may intrigue older adults’ interest in walking even though they were a bit further from their residences. Villanueva et al. [35] suggested that a greater distance to specific reactional destinations such as parks may still be attractive for older adults to walk for recreation, compared with walking for transport to get to and from other destinations. A previous study additionally showed that proximity to green spaces would be associated with a higher likelihood of walking maintenance over time [26]. Future research paying attention to the differences across buffer sizes for specific destinations or parks with specific attributes is required to corroborate these potential explanations.

Regarding all-day and time-specific PA and sedentary time, the results found that an increase in the number of parks within the residential neighbourhood was associated with more PA, except MVPA, and less sedentary time in the afternoon and evening but not morning. The findings may imply the time older adults likely travel and visit the park and engage in PA, which provides alternative activities to reduce indoor sedentary time in the afternoon and evening rather than in the morning. Previous studies from the US that observed people’s park use and park-based physical activity reported the lowest number of visits to parks in the morning, compared with that in the afternoon and evening [6, 24]. Thus, the amounts of PA accumulated within or around the parks may be the lowest in the morning than in the afternoon and evening. However, a previous study from Taipei, Taiwan, observed that older adults are more likely to be present in the parks in the morning rather than in the afternoon; those who visit the parks in the morning mainly attend organised activities [21]. It is an interesting finding implying that the diurnal pattern of PA related to parks may change during the pandemic in Taipei’s older adults. Before the COVID-19 pandemic (2011), the observations for park visits suggested that the primary purpose for older adults to visit parks in the morning was to participate in organised activities such as informal classes in dancing and martial arts [21]. By contrast, this study suggests that older adults may visit parks in the afternoon and evening to engage in unorganised activities due to the advocacy of social distancing and fear of the spread of the virus during the COVID-19 pandemic (2020–2021). Future studies can examine the argument by comparing older adults’ park visits between pre-pandemic, pandemic, and post-pandemic periods.

A lack of associations was found between the number of parks in the residential neighbourhood and MVPA time (except during the evening for the 800m buffer). Some previous studies also found no or weak associations between the number of parks and MVPA [4, 8, 24]. The lack of associations for MVPA may be attributable to fewer older adults engaged in vigorous-intensity PA in parks [33]. By contrast, a previous Dutch study reported positive associations between the number of parks within 1,000m and 2,000m and MVPA levels, although no longitudinal associations were found between park proximity and changes in MVPA levels over time [22]. Future observations into the intensity and type of physical activity were engaged in parks for older adults are required.

### Strengths and limitations

This is the first study to investigate the association of the number of parks with physical activities and sedentary time differs by time of the day using a sample of community-dwelling older population. The exposure and outcomes were objectively measured using GIS and an accelerometer. This study has several limitations. First, older adult participants were recruited from outpatient clinics and community check-ups based in one hospital in Taipei, Taiwan, and such recruitment may limit the generalisability of the results. Future studies are suggested to include a larger sample size of older adults across diverse areas. Furthermore, the recruitment period was during the COVID-19 pandemic. The advocacy of social distancing and fear of the spread of the virus may impact older adults’ diurnal pattern of outdoor physical activities. Second, the venue where physical activities and sedentary time occurs was not obtained. The mismatch between the venues for the exposure and outcomes may lead to a misinterpretation of parks due to the contribution of physical activities and sedentary time in a non-park context to total step counts, PA, and sedentary time. For instance, a previous study showed that the number of public open spaces within a 1 km residential buffer was negatively associated with all-day walking to public open spaces but not with the amount of walking within the public open spaces [14]. Further studies are suggested adding information on the global positioning system to identify the physical activities within and outside the parks. Third, we examined the number of parks within the neighbourhood but did not assess the attached facilities within the parks. Different types of facilities within the parks may have different impacts on diverse users. Fourth, the study was a cross-sectional design and therefore the causal relationships between the number of parks and behaviours cannot be directly inferred.

## Conclusions

The results of this study suggest that allocating parks not exceeding an 800m buffer of older adults’ residences can increase their physical activity and reduce sedentary time, particularly during the afternoon and evening. Public and social policies on urban planning of open public spaces that can promote physically-active behaviours and reduce sedentary time should consider the location with proper travel distance between the residence and parks. Future research using the longitudinal design is required to corroborate these associations.

## Data Availability

Data used in this study are available from the corresponding authors upon reasonable request.
